# Joint Multidimensional Pattern for Spectrum Prediction Using GNN

**DOI:** 10.3390/s23218883

**Published:** 2023-11-01

**Authors:** Xiaomin Wen, Shengliang Fang, Zhaojing Xu, Han Liu

**Affiliations:** 1 Graduate School, Space Engineering University, Beijing 101416, China; eeiwxm@163.com (X.W.);; 2 School of Space Information, Space Engineering University, Beijing 101416, China; 3 8511 Research Institute of China Aerospace Science & Industry Corp., Nanjing 210007, China

**Keywords:** graph convolutional neural networks, long short-term memory, power spectrum prediction, tensor graph

## Abstract

In general, judging the use/idle state of the wireless spectrum is the foundation for cognitive radio users (secondary users, SUs) to access limited spectrum resources efficiently. Rich information can be mined by the inherent correlation of electromagnetic spectrum data from SUs in time, frequency, space, and other dimensions. Therefore, how to efficiently use the spectrum status of each SU implementation of reception multidimensional combination forecasting is the core of this paper. In this paper, we propose a deep-learning hybrid model called TensorGCN-LSTM based on the tensor data structure. The model treats SUs deployed at different spatial locations under the same frequency, and the spectrum status of SUs themselves under different frequencies in the task area as nodes and constructs two types of graph structures. Graph convolutional operations are used to sequentially extract corresponding spatial-domain and frequency-domain features from the two types of graph structures. Then, the long short-term memory (LSTM) model is used to fuse the spatial, frequency, and temporal features of the cognitive radio environment data. Finally, the prediction task of the spectrum distribution situation is accomplished through fully connected layers. Specifically, the model constructs a tensor graph based on the spatial similarity of SUs’ locations and the frequency correlation between different frequency signals received by SUs, which describes the electromagnetic wave’s dependency relationship in spatial and frequency domains. LSTM is used to capture the electromagnetic wave’s dependency relationship in the temporal domain. To evaluate the effectiveness of the model, we conducted ablation experiments on LSTM, GCN, GC-LSTM, and TensorGCN-LSTM models using simulated data. The experimental results showed that our model achieves better prediction performance in RMSE, and the correlation coefficient R^2^ of 0.8753 also confirms the feasibility of the model.

## 1. Introduction

Due to the rapid development of modern wireless communication technology, various new wireless mobile terminals are emerging, and the demand for electromagnetic spectrum resources is increasing rapidly. Currently, wireless transmission services have been allocated in all frequency bands, and the spectrum resources are almost exhausted [1]. However, while the scarcity of spectrum resources is increasing, the problem of inefficient utilization and idleness of static spectrum resource management solutions is also very prominent [2]. Therefore, how to maximize the utilization of spectrum resources is currently an urgent problem that needs to be addressed.

Currently, cognitive radio networks (CRNs), which include key technologies such as dynamic spectrum access (DSA) and opportunistic spectrum access (OSA), are recognized as effective tools for improving the utilization of limited spectrum resources. CRNs perceive, recognize, and utilize the available spectrum in specific task spaces through self-learning and interaction with the surrounding environment, adapting to the constantly changing radio environment. Furthermore, the acquisition of spectrum status information by SUs through spectrum sensing is not only the first step in implementing CRNs but also the foundation for subsequent effective analysis and utilization of idle spectrum resources [3]. However, in practice, SUs often encounter problems such as long delays, high energy consumption, and limited capture range when scanning and sensing the entire spectrum (especially in wideband spectrum sensing tasks), which inevitably hinder the efficient operation of the CRN system [4].

To address the aforementioned problems, researchers have proposed spectrum prediction techniques. SUs can predict the future slot’s received spectrum power by mining and analyzing historical spectrum sensing data and then only sense the spatiotemporal spectrum resources with predicted values below the access power threshold, effectively reducing the time delay and energy consumption of subsequent processing. Early spectrum prediction research mainly focused on time-domain spectrum prediction methods, lacking research on multiple dimensions such as time, frequency, and space, including linear regression models [5], time series prediction models [6], Markov prediction models [7], neural network models [8], etc. The authors of [9] used the multiple attribute decision-making (MADM) methods and artificial neural network architecture to determine the best candidate channel to realize spectrum switching decisions. References [10,11] proposed using fuzzy decision-making principles to estimate handoff spectrum probability, which effectively improved switching efficiency. In recent years, deep learning models have become a powerful tool for spectrum prediction to leverage the potential correlations of frequency data in multiple dimensions such as time, frequency, and space [12,13,14].

In response to the spatial dependence, time dependence, or spectral dependence of spectrum data, composite neural networks such as long-short term memory (LSTM) models and convolutional neural networks (CNN) [15] have been used for joint spectrum prediction in multiple dimensions. Yu et al. [16] proposed a hierarchical dual-CNN and GRU (DCG) model for predicting the local spectrum availability of SUs in CR communication, which can explore the spectral and temporal correlations between spectrum occupancy data. However, simply connecting RNN and CNN still cannot build a comprehensive ability to discover correlations between spatiotemporal multidimensional input data. Reference [17] used transfer learning models for spectrum prediction, but due to the differences in frequency band data, prediction models cannot be directly used across frequency bands. The STS-PredNet [18] models that the received signal strength at a specific spatial location are determined by a weighted linear combination of multiple SUs. The weighting coefficients are obtained using the inverse distance weighting method based on the distance between SUs and the specific spatial location, which ignores the inherent spatial correlation between different observation locations. Model TF^2^AN [19] based on the preprocessing of the spectrum map, a weighted transfer learning model is introduced to share the spectrum knowledge among multiple locations and frequency bands to improve the performance of the spectrum prediction model. The input of the model SAE-TSS [20] is the image format, and the spectrum sequence is converted into the image format for offline training. The above models are based on the spectral data in space, time, and frequency domains, and make use of the complex correlation between cross-domain knowledge. However, they do not remove the influence of regular data structure and can not extract the inherent correlation of non-Euclidean space well enough. Recently, tensor analysis has been adopted as a framework [21,22] to leverage multidimensional correlations for spectrum prediction. However, using tensor decomposition to handle high-dimensional data requires a long computation time, and to achieve the highest possible prediction accuracy, it also requires the transmission of as much information as possible from the base station.

To address the above issue, this paper proposes a TensorGCN-LSTM hybrid network model to provide an effective method based on mining the implicit rules among electromagnetic data in the spatial, frequency, and temporal domains for cognitive radio task area. More specifically, the proposed approach considers SUs at different spatial locations and the spectrum states of SUs at different frequencies in the task area as nodes and constructs two categories of graph structures accordingly. Tensor graph convolution (TensorGCN) [23] is an effective structure for processing tensor data, which we introduce into the field of spectrum prediction to handle tensor graphs consisting of the two aforementioned graph structures. The essence of the TensorGCN-LSTM model is to utilize graph convolution operations to sequentially discover the correlation rules of spectrum data in the spatial and frequency domains, as well as use LSTM to explore the correlation rules in the temporal domain, thereby improving the accuracy of predicting the change in spectrum state over time and providing a basis for spectrum resource planning and scheduling. Comparative experimental results show that the TensorGCN-LSTM model can provide stable and accurate prediction results.

In summary, our core contributions are three-fold:

1. We abstract SUs as nodes and transform the spectrum prediction task into a supervised learning task based on graph tensor structured data. From the existing research literature, we first introduce the concept of graph tensor data structures into the field of spectrum prediction;
2. To extract the correlation features of different frequency data over a period received by SUs, we regard the SU’s state of receiving data at different frequencies as nodes (called virtual nodes) and design the inter-frequency graph network structure to extract the frequency-domain correlation features of the spectrum;
3. We propose TensorGCN-LSTM, a new joint prediction model in the time, space, and frequency domains, which integrates multidimensional features of task area spectrum data to predict the spectrum state. Ablation experimental results show that compared with other single time-series prediction methods and spatiotemporal prediction methods, the TensorGCN-LSTM model has a more accurate prediction performance.

The rest of this paper is organized into four sections. Section 2 presents the preliminary works, including the establishment of the tensor graph and definition of the spectrum prediction. Section 3 describes the methodology of the deep learning model for forecasting spectrum evolution. Section 4 presents an introduction to the experiment settings and dataset. Section 5 discusses the evaluation of the results. Finally, concluding remarks and future research directions are discussed in Section 6. Section 7 introduces a patent resulting from the work reported in this paper.

## 2. Preliminaries

In this section, we preprocessed the received signal strength (RSS) of secondary users collected on various frequency bands over time in the cognitive radio task area to form a tensor graph signal. Based on the spatial and frequency domain states of secondary users, we established two types of graphs and composed a tensor graph model.

### 2.1. Establishment of Tensor Graph Signal

**Figure 1:**
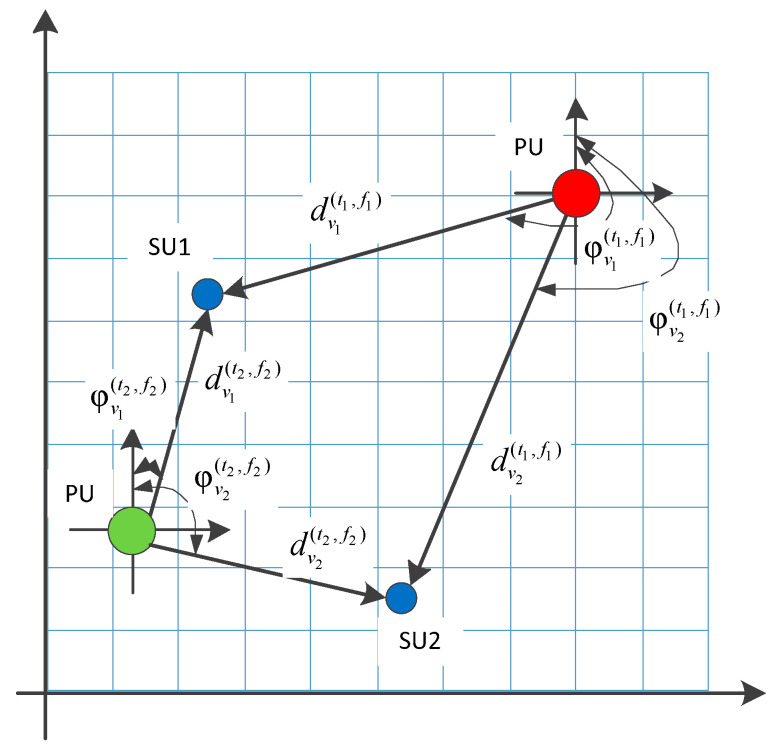
Illustration of node attribute characteristics (where red and green dots represent the location of the mobile primary user (PU) in two different time slots and working frequencies and blue dots represent different locations of secondary users (SUs) distributed in the wireless task area).

As illustrated in Figure 1, we divided the cognitive wireless task area into equidistant grids. For any secondary user node $v_{n},(n=1,2,\ldots ,N)$, the received signal strength, distance, and azimuth between the node and the mobile primary user at time slot t and working frequency $f_{k}$ are denoted as $\psi_{v_{n}}^{\left(t,f_{k}\right)}$, $d_{v_{n}}^{\left(t,f_{k}\right)}$, and $\varphi_{v_{n}}^{\left(t,f_{k}\right)}$, respectively. We can establish the feature vector of secondary users that varies with time and frequency:

(1) $$x_{{}_{v_{n}}}^{\left(t,f_{k}\right)}=\left[\psi_{v_{n}}^{\left(t,f_{k}\right)},d_{v_{n}}^{\left(t,f_{k}\right)},\varphi_{v_{n}}^{\left(t,f_{k}\right)}\right]\in {\mathbb{R}}^{1\times M}$$

where k=1,2,…,K and M represents the number of features of a secondary user node.

Based on the description of the features $x_{{}_{v_{n}}}^{\left(t,f_{k}\right)}$ of a single secondary user node, we can construct the features matrix $X^{\left(t,f_{k}\right)}$ from the data of N secondary user nodes $V={\left\{v_{n}\right\}}_{n=1}^{N}$ at time slot t and working frequency $f_{k}$:

(2) $$X^{\left(t,f_{k}\right)}=\left[x_{{}_{v_{1}}}^{\left(t,f_{k}\right)},x_{{}_{v_{2}}}^{\left(t,f_{k}\right)},\ldots ,x_{{}_{v_{N}}}^{\left(t,f_{k}\right)}\right]\in {\mathbb{R}}^{N\times M}$$

Therefore, the feature tensor of the graph signal $X^{\left(t\right)}$, which captures the data of N SUs at K different monitoring frequencies, can be constructed from $X^{\left(t,f_{k}\right)}$ as:

(3) $$X^{\left(t\right)}=\left[X^{\left(t,f_{1}\right)},X^{\left(t,f_{2}\right)},\ldots ,X^{\left(t,f_{K}\right)}\right]\in {\mathbb{R}}^{K\times N\times M}$$

### 2.2. Establishment of Tensor Graph

**Figure 2:**
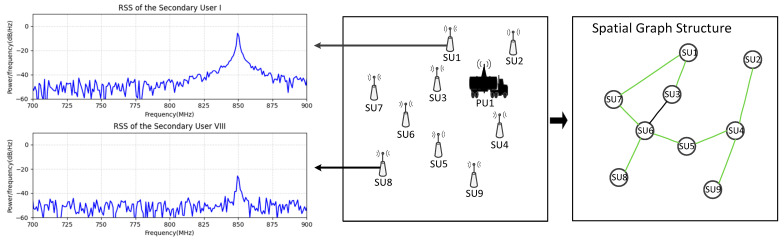
Establishment of the network graph structure by SUs.

We set up the problem of predicting the graph spectrum, as shown in the construction process in Figure 2. From left to right, the figure shows the power spectrum received by secondary users (SU) from monitoring, the spatial distribution of SUs and mobile primary users (PU) within the task area, and the network graph structure constructed by secondary users according to certain rules.

#### 2.2.1. The Spatial Domain Graph Structures

**Figure 3:**
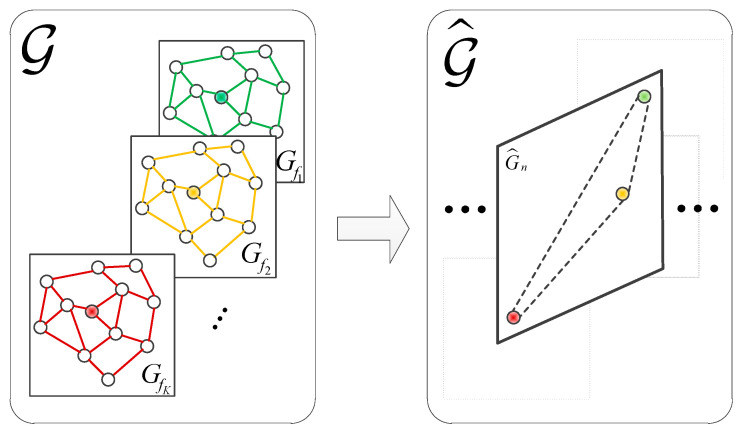
The construction of the spatial domain graph and the frequency domain graph. (The green, yellow, and brown dots represent one secondary user node at different monitoring frequencies. The left figure shows the spatial domain graph structures of all secondary user nodes at different monitoring frequencies, while the right figure illustrates the process of extracting frequency domain features of the secondary user node $v_{n}$ at different monitoring frequencies after completing spatial feature extraction in the left figure).

The graph of N SUs in the task area at monitoring frequency $f_{k}$ is shown in Figure 2 (right). It is referred to as a spatial domain graph structure, which is denoted $G_{f_{k}}=(V,A_{f_{k}})$ in Figure 3 (left). $A_{f_{k}}$ is the adjacency matrix that describes the spatial domain graph $G_{f_{k}}$, where each element represents the connectivity between nodes.

We assumed that the transmitting and receiving antennas in cognitive radio networks are both omnidirectional antennas. The formula for calculating the path loss (PL) of free space electromagnetic wave propagation is:

(4) $$PL={\left(\frac{4\pi •d}{\lambda }\right)}^{2}={\left(\frac{4\pi f•d}{c}\right)}^{2}$$

where λ,f, respectively, denote the wavelength and frequency of the PU’s transmission carrier. $c=3\times 10^{8}$ m/s. d denotes the distance between SUs and PUs. Therefore, the relationship between the received power $P_{r}$ of the secondary user’s receiver and the transmission power $P_{t}$ of the primary user is given by:

(5) $$P_{r}=\frac{P_{t}}{PL}$$

As can be seen from Equations (4) and (5), assuming a fixed transmission carrier frequency by the primary user, the critical factor affecting the RSS of the secondary user is the distance between the SU and the PU. Therefore, we adopted the “inverse distance weighting method” to construct the adjacency matrix $A_{f_{k}}$ of the spatial domain graph, as follows:

(6) $$A_{f_{k}}=\left[\begin{array}{ccccc} a_{1,1} & \ldots & a_{1,j} & \ldots & a_{1,N}\\ \vdots & \ddots & \vdots & \ddots & \vdots \\ a_{i,1} & \ldots & a_{i,j} & \ldots & a_{i,N}\\ \vdots & \ldots & \vdots & \ddots & \vdots \\ a_{N,1} & \ldots & a_{N,j} & \ldots & a_{N,N} \end{array}\right]$$

where $a_{ij}=\{\begin{array}{cc} \frac{1}{d(v_{i},v_{j})}, & i\neq j\mathrm{and}d(v_{i},v_{j})<th\\ 0, & \mathrm{otherwise} \end{array}$, $d(v_{i},v_{j})$ denote Euclidean distance between the node $v_{i}$ and $v_{j}$. th represents the Euclidean distance threshold for establishing edges between nodes.

#### 2.2.2. The Frequency Domain Graph Structures

In this section, we constructed a graph structure for the state relationships of a node $v_{n}$ when receiving data at different frequencies, which is called the “frequency domain graph structure”. We refer to the spectrum state corresponding to each frequency signal received by the SU node as a “virtual node”. $v_{n,f_{k}}=\left[\psi_{v_{n}}^{\left(t-\Delta t+1,f_{k}\right)},\cdots ,\psi_{v_{n}}^{\left(t,f_{k}\right)}\right]$ denotes the spectrum state of the secondary user node $v_{n}$ when continuously receiving data for Δt time slots at a certain frequency. ${\hat{V}}_{n}={\left\{v_{n,f_{k}}\right\}}_{k=1}^{K}$ denotes a collection of virtual nodes at K frequencies. The elements of the adjacency matrix ${\hat{A}}_{n}\in {\mathbb{R}}^{K\times K}$ defined in Equation (8) represent the frequency domain similarity between virtual nodes from SU $v_{n}$. Therefore, the frequency domain graph structures can be denoted as ${\hat{G}}_{n}=\left({\hat{V}}_{n},{\hat{A}}_{n}\right)$ shown in Figure 3 (right).

In the frequency domain graphs, we use correlation coefficients to analyze the inherent frequency domain correlation about the measured spectrum data:

(7) $$\rho_{f_{k},f_{l}}=\frac{\mathrm{cov}(v_{n,f_{k}},v_{n,f_{l}})}{\sigma_{v_{n,f_{k}}}\sigma_{v_{n,f_{l}}}}=\frac{E\left[\left(v_{n,f_{k}}-\mu_{v_{n,f_{k}}})(v_{n,f_{l}}-\mu_{v_{n,f_{l}}}\right)\right]}{\sigma_{v_{n,f_{k}}}\sigma_{v_{n,f_{l}}}}$$

In Equation (7), cov(•) represents the covariance operator, while μ and σ, respectively, represent the mean value and standard deviation. The closer the absolute value $\rho_{f_{k},f_{l}}\in \left[-1,1\right]$ is to 1, the stronger the correlation between the two frequencies $f_{k}$ and $f_{l}$ of secondary user $v_{n}$ during Δt. The adjacency matrix ${\hat{A}}_{n}\in {\mathbb{R}}^{K\times K}$ can be defined as follows:

(8) $${\hat{A}}_{n}=\left[\begin{array}{ccccc} {\hat{a}}_{1,1} & \ldots & {\hat{a}}_{1,l} & \ldots & {\hat{a}}_{1,K}\\ \vdots & \ddots & \vdots & \ddots & \vdots \\ {\hat{a}}_{k,1} & \ldots & {\hat{a}}_{k,l} & \ldots & {\hat{a}}_{k,K}\\ \vdots & \ldots & \vdots & \ddots & \vdots \\ {\hat{a}}_{K,1} & \ldots & {\hat{a}}_{K,l} & \ldots & {\hat{a}}_{K,K} \end{array}\right]$$

where ${\hat{a}}_{k,l}=\{\begin{array}{cc} \rho_{f_{k},f_{l}} & v_{n,f_{k}}↔v_{n,f_{l}}\mathrm{and}k\neq l\\ 0 & \mathrm{otherwise} \end{array}$. Furthermore, a tensor graph can be constructed as $G=\left[{\left[G_{f_{k}}\right]}_{k=1}^{K},{\hat{G}}_{n}\right]$.

### 2.3. The Definition of the Spectrum Prediction Problem

According to the above description, the graph tensor signal received by SUs during $\left[t-T^{\prime }+1,t\right]$ in the cognitive radio task area can be represented as $\left[X^{\left(t\right)},\ldots ,X^{\left(t-T^{\prime }+1\right)}\right]$. The received RSS by a secondary user in the next T time slots can be represented as $\Psi_{n}\left(f,t\right)={\left\{\psi_{v_{n}}^{\left(t,f\right)}\right\}}_{n=1}^{N}$, where $f={\left\{f_{k}\right\}}_{k=1}^{K}$, t∈[t+1,t+T]. Therefore, the graph tensor G and graph tensor signal $X^{\left(t\right)}$ are learned by the proposed composite deep neural network model TensorGCN-LSTM to obtain a mapping function F, denoted as Equation (9), which predicts future spectral data using historical spectrum data. Moreover, the model is enabled to implement the prediction of spectrum evolution.

(9) $$\Psi_{n}\left(f,t\right)=F(X^{\left(t\right)},\ldots ,X^{\left(t-T^{\prime }+1\right)},G)$$

## 3. Methodology

**Figure 4:**
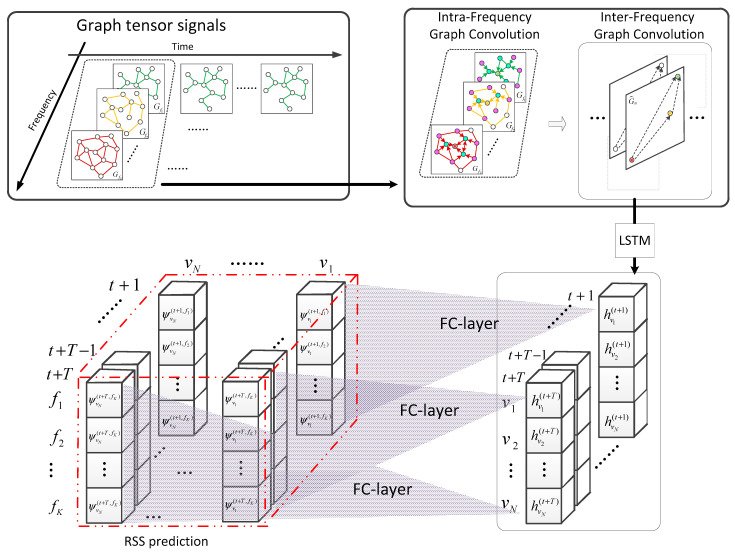
Schematic diagram of the TensorGCN-LSTM spectrum prediction model. (The green, orange, and brown nodes in $G_{f_{1}}$, $G_{f_{2}}$ and $G_{f_{k}}$ respectively represent the status of the same node at different operating frequencies. The blue and pink nodes in the upper-right sub-graph represent the first-order and second-order neighboring nodes, respectively. In the lower left figure, the three-dimensional data cube is composed of node–frequency–time, which can visually represent the RSS of secondary user nodes at specific frequencies and time points).

In this section, we elaborate on the implementation process of the prediction method based on the TensorGCN-LSTM hybrid model, shown in detail in Figure 4. The model first performs graph convolution on the node features in the spatial domain graph structure to generate node embedding. Then, it combines the node embedding with the spectral graph structure and performs secondary graph convolution to extract information that integrates spatial and spectral information from secondary users. We refer to the above two graph convolution operations as intra-frequency graph convolution and inter-frequency graph convolution, respectively. They are shown in Figure 4 (upper right). Afterward, the spatial–spectral embeddings are fed into the LSTM model to generate fusion feature information in multiple dimensions of spatial, spectral, and temporal domains. Finally, the fusion features are passed through a fully connected layer to output the predicted RSS results.

According to the processing method adopted by the graph convolutional neural network [24], the forward propagation formula of the graph convolution for US nodes in the spatial domain graph structure is as follows:

(10) $$H_{v_{n}}^{\left(t,f_{k}\right)}=\sum_{r=1}^{R}P_{r}(\tilde{L})X_{v_{n}}^{\left(t,f_{k}\right)}\Theta_{r}$$

where $\Theta_{r}\in {\mathbb{R}}^{M\times W}$ is the parameter matrix of the filter for intra-frequency graph convolution that needs to be learned and updated. $P_{r}(\tilde{L})\in {\mathbb{R}}^{N\times N}$ is the *r*-th order Chebyshev polynomial and the standardized Laplace matrix $\tilde{L}$ of adjacency matrix $A_{f_{k}}$ refers to:

(11) $$\tilde{L}=\frac{2(I-D^{-\frac{1}{2}}A_{f_{k}}D^{-\frac{1}{2}})}{\lambda_{\max}}-I$$

where $\lambda_{\max}$ represents the maximum eigenvalue of the Laplacian matrix $\tilde{L}$. I and, respectively, refer to the identity matrix and degree matrix of the matrix $A_{f_{k}}$.

$H\in {\mathbb{R}}^{1\times W}$ in Equation (10) is the spatial embedding vector extracted by graph convolution. Therefore, ${\hat{X}}_{v_{n}}^{\left(t\right)}=\left[H_{v_{n}}^{\left(t,f_{1}\right)},H_{v_{n}}^{\left(t,f_{2}\right)},\ldots ,H_{v_{n}}^{\left(t,f_{K}\right)}\right]$ serves as the feature matrix for input inter-frequency graph convolution, and the input vector $y_{v_{n}}^{\left(t\right)}$ to the LSTM module can be obtained through the following:

(12) $$y_{v_{n}}^{\left(t\right)}=\sum_{r^{\prime }=1}^{R^{\prime }}P_{r^{\prime }}(\tilde{L^{\prime }}){\hat{X}}_{v_{n}}^{\left(t\right)}{\Theta^{\prime }}_{r^{\prime }}$$

Similar to Equation (10), $\tilde{L^{\prime }}$ represents the normalized Laplacian matrix corresponding to the adjacency matrix ${\hat{A}}_{n}\in {\mathbb{R}}^{K\times K}$. ${\Theta^{\prime }}_{r^{\prime }}$ is the filter parameter matrix that needs to be learned and updated through inter-frequency graph convolution. $P_{r^{\prime }}(\tilde{L^{\prime }})\in {\mathbb{R}}^{K\times K}$ is the Chebyshev polynomial of $r^{\prime }$ order. It should be noted that due to the filtering operation being an approximation of the *R*-th order Laplacian operator, it is localized to *R*-order neighboring nodes. In our experiments, we set $R=R^{\prime }=2$.

**Figure 5:**
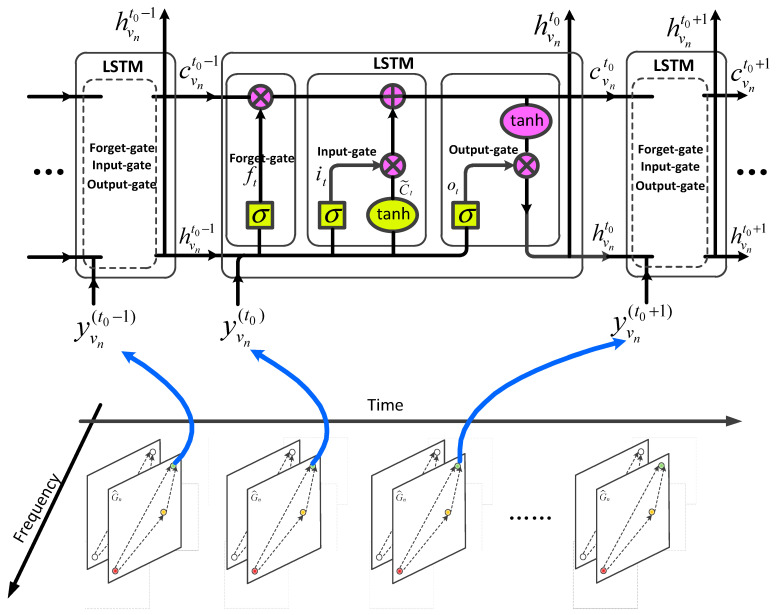
Diagram of inputting the fused spatial–frequency domain feature into the LSTM model.

To learn the temporal evolution characteristics of electromagnetic waves, we input the fused spatial and frequency domain embedding $y_{v_{n}}^{\left(t\right)}$ of each secondary user node into an LSTM model [25]. This operation is shown in Figure 5. 

At each time slot, the LSTM unit takes the fused embedding $y_{v_{n}}^{\left(t\right)}$ of the node as input, which enables the LSTM model to more comprehensively describe the temporal evolution process of electromagnetic waves based on the integrated frequency and spatial propagation characteristics. We describe the entire process of the LSTM using Equation (13):

(13) $$\begin{array}{l} f_{v_{n}}^{t}=\sigma \left(W_{f}•\left[h_{v_{n}}^{t-1},y_{v_{n}}^{\left(t\right)}\right]+b_{f}\right)\\ i_{v_{n}}^{t}=\sigma \left(W_{i}•\left[h_{v_{n}}^{t-1},y_{v_{n}}^{\left(t\right)}\right]+b_{i}\right)\\ {\tilde{C}}_{v_{n}}^{t}=\tanh\left(W_{c}•\left[h_{v_{n}}^{t-1},y_{v_{n}}^{\left(t\right)}\right]+b_{c}\right)\\ c_{v_{n}}^{t}=f_{v_{n},f_{k}}^{t}\circ c_{v_{n}}^{t-1}+i_{v_{n}}^{t}\circ {\tilde{C}}_{v_{n}}^{t}\\ o_{v_{n}}^{t}=\sigma (W_{o}•\left[h_{v_{n}}^{t-1},y_{v_{n}}^{\left(t\right)}\right]+b_{o})\\ h_{v_{n}}^{t}=o_{v_{n}}^{t}\circ \tanh(c_{v_{n}}^{t}) \end{array}$$

Based on the output of the proposed TensorGCN-LSTM model, we finally predict RSS by:

(14) $$\left[\psi_{v_{n}}^{\left(t+l,f_{1}\right)},\psi_{v_{n}}^{\left(t+l,f_{1}\right)},\ldots ,\psi_{v_{n}}^{\left(t+l,f_{1}\right)}]=FC(h_{v_{n}}^{t}\right)$$

where ${\left\{\psi_{v_{n}}^{\left(t+l,f_{i}\right)}\right\}}_{k=1}^{K}$ denote the RSS of SU $v_{n}$ corresponding to K frequencies at the time t+l in the future and FC(•) is a full connection layer.

## 4. Numerical Experiments

The datasets of the simulation experiment were generated based on the addition of mobile transmitters with random emission frequencies. The position coordinates of the considered transmitters varied randomly and uniformly with time. The lognormal shadowing model adhered to the Gudmundson model [26], which provides the correlation between the PU and SUs. Multiple mobile primary users working on different frequencies were added to the cognitive radio task region, forming experimental data of power spectral density with spatial and frequency domain characteristics that continuously varied over time. 

For simplicity, the transmission power of each primary user was set at 1 w. In addition, for the representation of temporal data, we uniformly divided the time axis into windows and aggregated the spectral data within the same time window Δt into one-time steps. Finally, we used discretized time steps to represent continuous temporal data.

### 4.1. Experiment Settings

**Figure 6:**
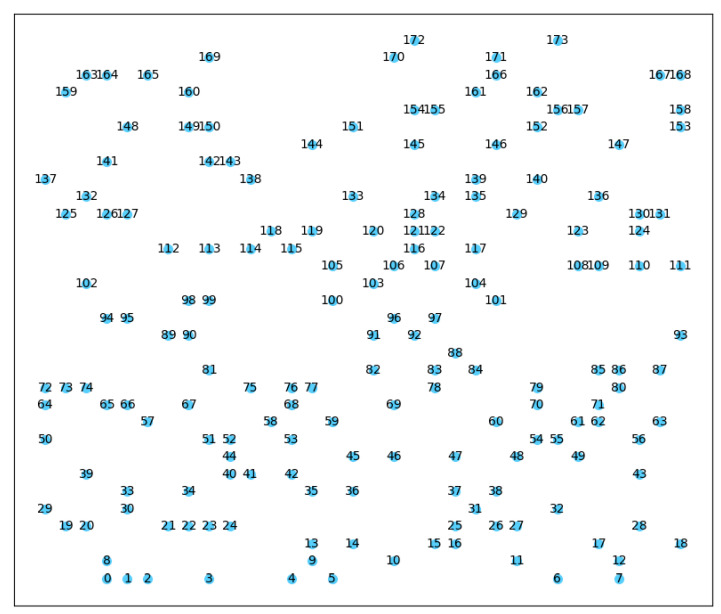
Spatial distribution map of secondary users. (We number the secondary users (0–173) in the cognitive radio task region and highlighted them in blue for clarity).

According to the spatial resolution requirements of the spectrum prediction task, we divided the cognitive radio task region into a 200 × 200 grid and randomly distributed 174 secondary users uniformly in each grid, as shown in Figure 6. The monitored frequency range was between 800 and 900 MHz, with a frequency resolution and spectrum sensing sweep span of 200 kHz for the spectrum sensor, generating a total of 500 frequency bands.

In the experiment, under the premise of examining whether the prediction model worked and not caring about the accuracy of radio wave propagation attenuation, we only considered the path loss and shadow fading of radio wave propagation for the attenuation of the spectrum sensor’s received power.

For the simulation experiment, the log-normal shadow fading model ($\mu_{sdw}=0$, $\sigma_{sdw}=0.5$) was used to model the shadow fading of the task area. The path propagation loss in the task area was modeled using a logarithmic distance path loss model, which is shown in Equation (15):

(15) $$P_{r}\left(\mathrm{dBm}\right)=P_{t}\left(\mathrm{dBm}\right)+K\left(\mathrm{dB}\right)-10\gamma \log_{10}\frac{d}{d_{0}}$$

where K is a constant coefficient related to the gain of the transmitting antenna, which is generally represented by the measured power value at $d_{0}$. Here, $d_{0}$ represents the far field distance of the antenna and is a constant reference distance. d is the distance between the receiver of the secondary user and the transmitter of the primary user. In the simulation experiment, we gridded the target area and set $d_{0}=1$ to represent the path loss of radio wave propagation attenuated by each grid. γ is the path loss exponent, which typically ranges from 3.7 to 6.5 for urban macrocells. In the experiment, γ was set to 5.

### 4.2. Dataset Preparation

**Figure 7:**
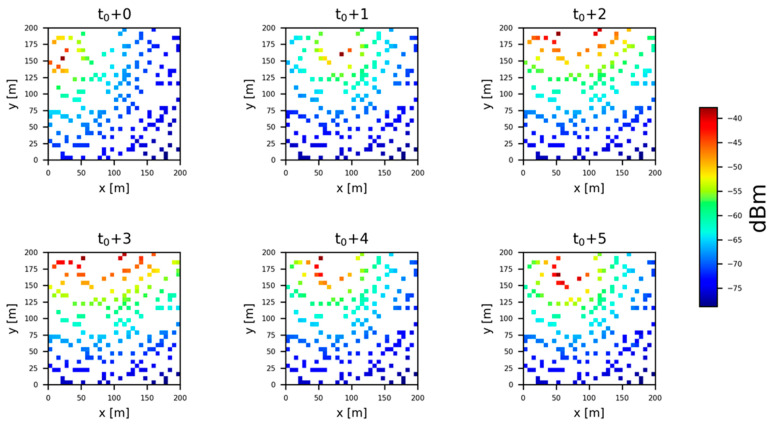
Time-series plot of RSS distribution.

We had each secondary user collect spectrum data for each frequency band in the task area over 17,280 time slots. We then divided the dataset into the training set, validation set, and test set in a ratio of 6:2:2. Figure 7 shows the RSS distribution of the task area for 174 secondary users continuously receiving 6 time-slots at 800 MHz.

**Figure 8:**
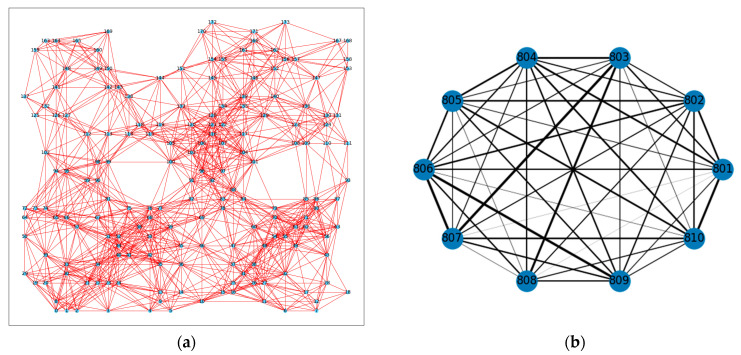
(**a**) Graph structure of the spatial domain; (**b**) Graph structure of the frequency domain.

Following the description in Section 2.2, we constructed spatial- and frequency-domain structure diagrams. As shown in Figure 8a, the spatial-domain structure was constructed for 174 secondary user nodes at different frequencies. The coordinate positions of each node in the diagram corresponded to the spatial coordinates of the secondary users in the task area. To concisely represent the frequency-domain structure, we selected a schematic diagram of the frequency domain graph for 10 frequencies (801 MHz, …, 810 MHz) within the 800–810 MHz frequency range. Each frequency state was treated as a node, and the absolute value of the correlation coefficient between the spectrum data of each frequency was used as the weight for the corresponding edge. This allowed us to construct a graph structure with frequencies as nodes, as shown in Figure 8b.

## 5. Discussion

**Figure 9:**
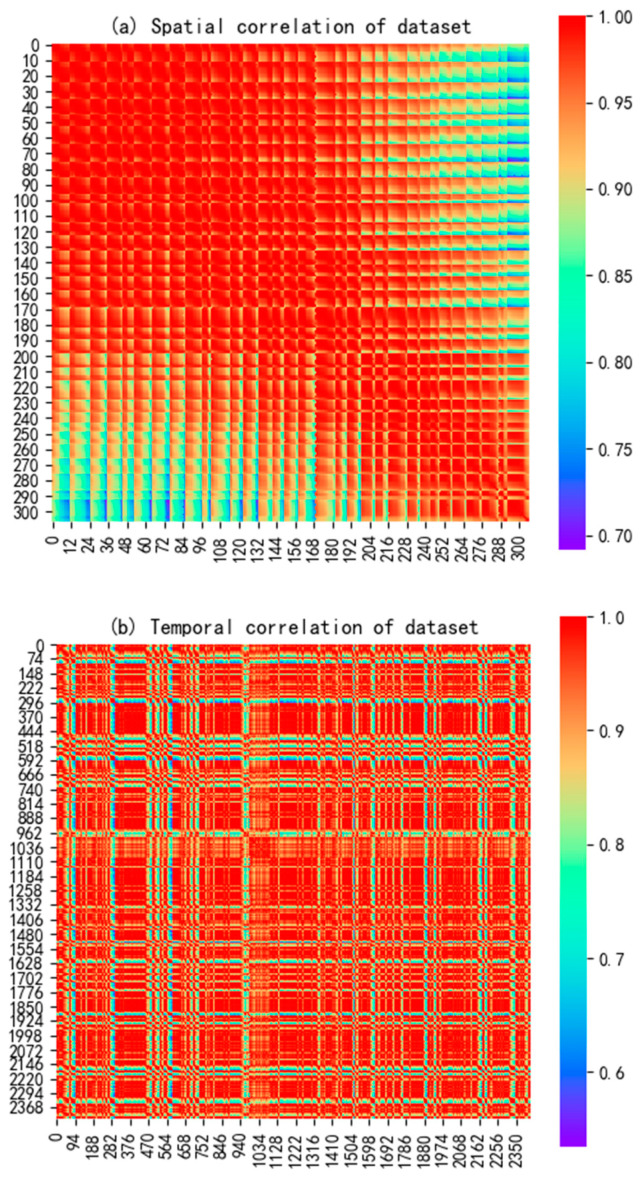

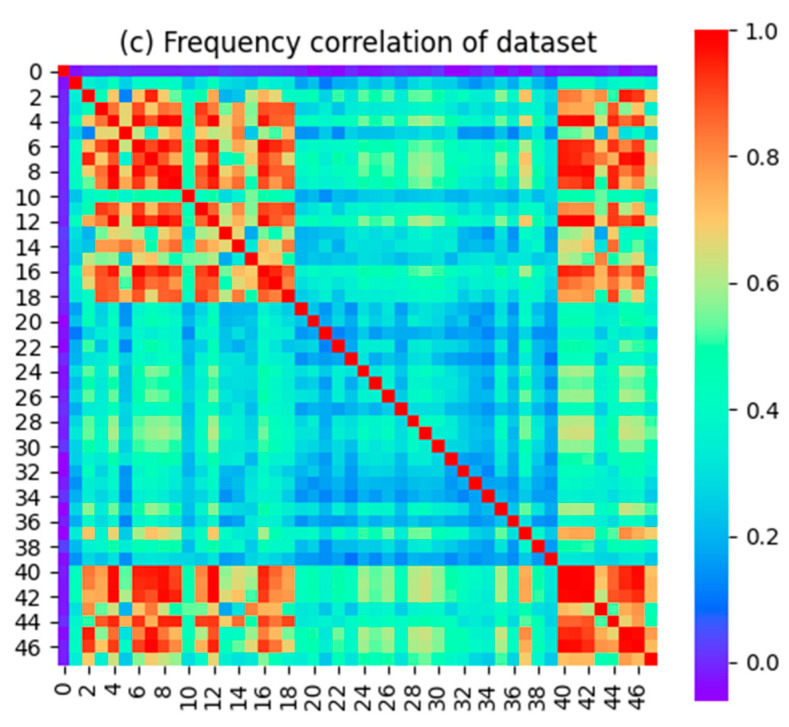
Correlation verification results of simulated datasets.

To validate the feasibility of conducting power spectral data prediction experiments using simulated datasets, we calculated the data correlations of individual secondary users in the time, frequency, and spatial domains, as shown in Figure 9. Specifically, Figure 9a illustrates the spatial correlation structure among secondary users, indicating a strong spatial correlation among them. Moreover, the proximity of secondary user indices reflected a stronger spatial correlation between closely located secondary users. In Figure 9b, the time-domain correlation of power spectral data for the same secondary user node across different frequency bands is depicted. It can be observed that the values of time-domain correlation were generally large, and the correlation distribution graph demonstrated the regularity of tidal effects in the spatial activity of the primary user. Figure 9c presents the distribution of frequency-domain correlations between any two time slots of the spectral state on a sensing node in the simulated dataset. Although the numerical values of frequency-domain correlation might not be as close to 1 as those of spatial and time-domain correlation, there were still some significant correlation values in certain frequency bands. The occurrence of windowing effects in the 48 frequency points within the frequency range of 800 to 810 MHz indicated a highly correlated spectral state evolution between low-frequency and high-frequency bands.

To validate the effectiveness of the proposed TensorGCN-LSTM model, we conducted experimental comparisons with three other models: LSTM, GCN, and GC-LSTM. We evaluated the generalization ability of each model by analyzing the loss values on the training, validation, and test sets. Additionally, we examined the prediction accuracy of the models using metrics such as the MAE (Mean Absolute Error), RMSE (Root Mean Square Error), and $R^{2}$ (coefficient of determination). The calculations were performed according to Equation (16):

(16) $$\begin{array}{l} MAE=\frac{\sum_{t=1}^{T}\left|\psi_{v_{n}}^{\left(t\right)}-{\hat{\psi }}_{v_{n}}^{\left(t\right)}\right|}{T}\\ RMSE=\sqrt{\frac{\sum_{t=1}^{T}{\left(\psi_{v_{n}}^{\left(t\right)}-{\hat{\psi }}_{v_{n}}^{\left(t\right)}\right)}^{2}}{T}}\\ MAPE=\frac{1}{T}\sum_{t=1}^{T}\left|\frac{\psi_{v_{n}}^{\left(t\right)}-{\hat{\psi }}_{v_{n}}^{\left(t\right)}}{\psi_{v_{n}}^{\left(t\right)}}\right|*100\%\\ R^{2}=1-\frac{\sum_{t=1}^{T}{\left(\psi_{v_{n}}^{\left(t\right)}-{\hat{\psi }}_{v_{n}}^{\left(t\right)}\right)}^{2}}{\sum_{t=1}^{T}{\left(\psi_{v_{n}}^{\left(t\right)}-{\bar{\psi }}_{v_{n}}\right)}^{2}} \end{array}$$

where $\psi_{v_{n}}^{\left(t\right)}$ and ${\hat{\psi }}_{v_{n}}^{\left(t\right)}$ represent the true values and predicted values of RSS, respectively. T represents the number of received data samples from secondary users, and ${\bar{\psi }}_{v_{n}}$ represents the sample mean.

The evaluation results of the loss function metrics for each model were the average values of the predicted results from 174 secondary user nodes. The models were trained using 24 historical samples to predict the data for the next 30 time slots. Table 1 presents the average cumulative losses of the four prediction models on the training, test, and validation sets at a frequency of 810 MHz.

**Figure 10:**
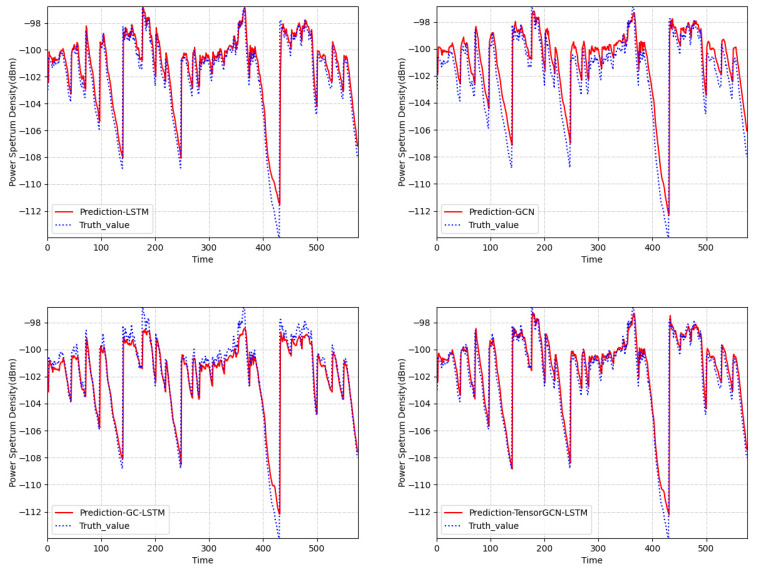
Comparison graph of predicted values and actual data curves of received signal strength for node 0 among four prediction models.

When evaluating the prediction error metrics of the prediction model, we conducted experimental comparisons using the RSS data received at 810 MHz frequency by the secondary user with index 0 ($v_{0}$) in the spatial domain graph structure shown in Figure 8a. To further explore the temporal variation in the ground-level RSS and four model predictions, we randomly selected the predicted results of 580 consecutive time slots for the secondary user $v_{0}$. Figure 10 displays the predicted power spectral density values of four models compared to the true power spectral density values. Generally speaking, the prediction curve of the TensorGCN-LSTM model aligned more closely with the actual trend and was closer to the real data. It can be seen that LSTM better grasped the changing trend of data in the time domain. Meanwhile, the spatial prediction model (GCN) showed a tendency to overestimate the ground-true value, and the spatiotemporal prediction model (GC-LSTM) showed an underestimation of the high values.

**Figure 11:**
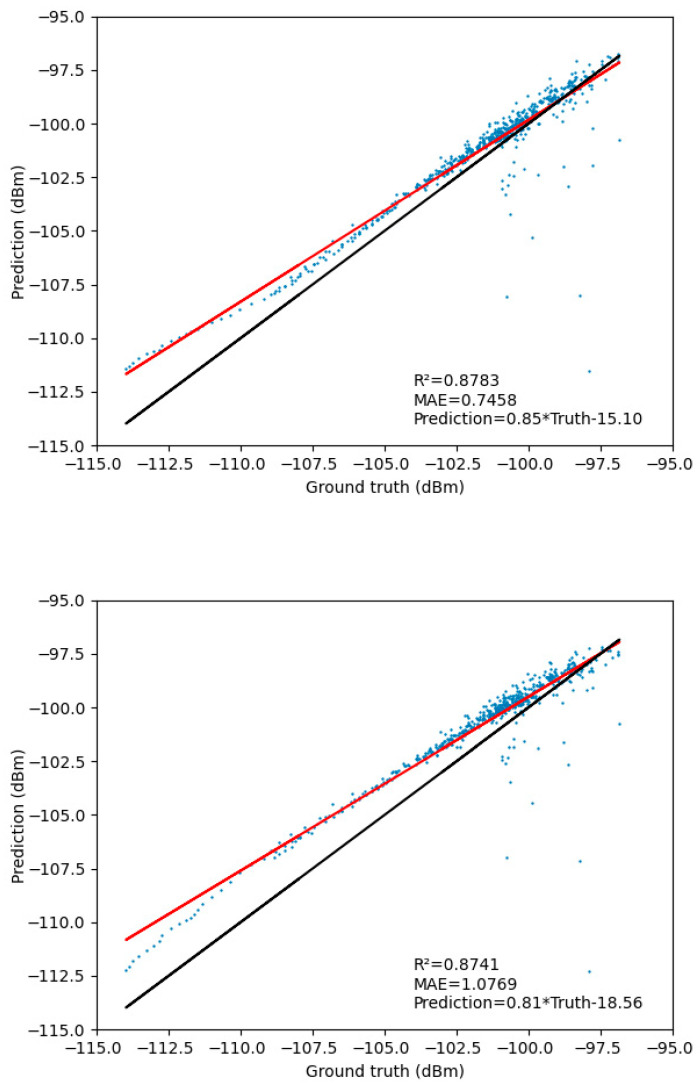

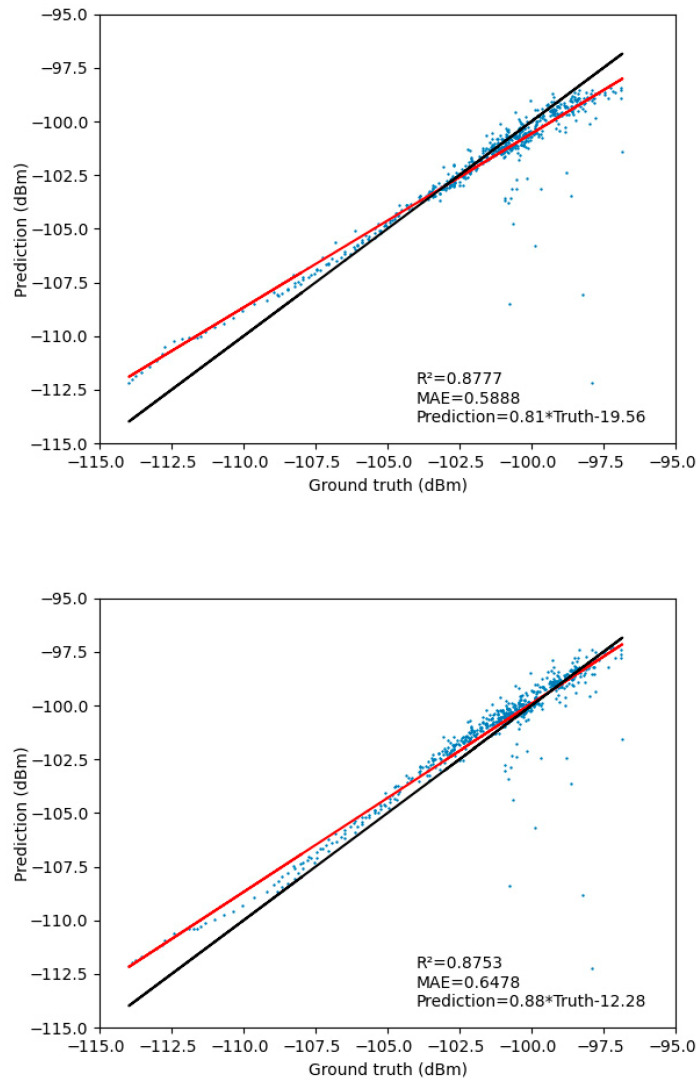
Comparison graph of predicted values and actual data curves of received signal strength for node 0 in Figure 6 among four prediction models. The diagonal line in the figure is determined by the equation: Prediction=a×Truth+b. Among them, a=1 and b=0 in the black diagonal line indicate that the predicted value is completely consistent with the measured value. The values of a and b in the red diagonal are given in the bottom right corner of each subfigure. The blue scatter points represent the predicted values of the models corresponding to the measured values.

In Figure 11, the Pearson linear correlation between the predicted and actual values revealed that, as the spatial, temporal, and frequency features fused, the predicted results exhibited a more concentrated numerical distribution with reduced variance. The slope corresponding to the TensorGCN-LSTM model (0.88) was less than one and the largest, indicating that our proposed model achieves a more balanced distribution trend between underestimation of low values and overestimation of high values. This strongly demonstrated that the fusion of multiple feature attributes contributes to the overall smoothness of the model’s predictions. Additionally, the *R*-value ($R^{2}=0.8753$) and *MAE* value (MAE=0.6478) of the TensorGCN-LSTM model indicated a strong consistency between the predicted values and the actual values. 

Table 2 presents a comparison of prediction errors for four prediction models under different prediction horizons. From the table, it can be observed that the TensorGCN-LSTM model exhibited varying degrees of reduction in prediction errors compared to the other three models, as indicated by RMSE, MAE, and MAPE metrics. The results demonstrated that considering the spatial and frequency distribution characteristics of radio propagation improves the prediction accuracy of the TensorGCN-LSTM model. Looking at the prediction error results of the GCN and LSTM models, it was evident that a neural network structure solely focusing on spatial correlations cannot effectively enhance the predictive accuracy of temporal data. With an increase in the prediction horizon, the uncertainty of all four models’ predictions increased, leading to gradually larger prediction errors. However, based on the comparison results for the 20th–30th horizons, our proposed TensorGCN-LSTM model exhibited better long-term prediction capability. This finding validates the beneficial effects of effectively integrating temporal-, spatial-, and frequency-domain features to enhance the prediction performance of the model.

The purpose of our experiment was to validate the effectiveness of the proposed model. The shadow fading component in the synthetic data generation followed the Gudmundson model, while the actual data were more complex than this. As a result, the complexity of the simulated experimental data may not be as high as that of real measurement data, and the variations in spectrum data may not be significant in the spatial and frequency domain. Consequently, the overall difference in error metrics among the four prediction models is not substantial. However, experiments on the simulated dataset have demonstrated that TensorGCN-LSTM exhibits significant potential in exploring the multidimensional interactions of spectrum prediction.

## 6. Conclusions

In this paper, we proposed a novel graph neural network deep learning framework called TensorGCN-LSTM for spectrum prediction. First, based on the global spatial distribution map of secondary users in the task area, we utilized the “spatial domain graph structure” to capture the characteristics of electromagnetic wave propagation in spatial space. Additionally, we employ the “frequency domain graph structure” to capture the frequency domain correlation between spectrum states in different service frequency bands. Subsequently, the LSTM model was used to summarize the temporal variation features of the secondary users’ network received power. Finally, by integrating the interaction information of spatial, frequency, and temporal domains through fully connected layers, we achieved the prediction of spectrum trends under the conditions of multi-dimensional information fusion. We showed the success of our approach through experiments on a simulated dataset that explored the multidimensional interactions of spectrum prediction. In future work, we plan to incorporate real measurement data and incorporate additional domain knowledge, such as terrain structures and weather information, to further enhance its accuracy and robustness in spectrum prediction.

## 7. Patents

There is a patent “A Prediction Method of Radio Environment Map [27]” resulting from the work reported in this manuscript.

## Figures and Tables

**Table 1:** Comparison of models’ losses on training, validation, and testing sets.

Metric	LSTM	GCN	GCN-LSTM	TensorGCN-LSTM
Train Loss	0.2055	0.3326	0.1674	0.1663
Validate Loss	0.2514	0.3521	0.1705	0.1501
Test Loss	0.2900	0.4601	0.1780	0.1483

**Table 2:** Comparison of prediction errors among four models when predicting RSS values for different time slot lengths. Best scores are in bold.

Horizon	LSTM			GCN			GC-LSTM			TensorGCN-LSTM		
	RMSE	MAE	MAPE	RMSE	MAE	MAPE	RMSE	MAE	MAPE	RMSE	MAE	MAPE
+1	1.1822	0.7458	0.73%	1.4020	1.0769	1.04%	1.1467	**0.5888**	**0.58%**	**1.1447**	0.6078	0.63%
+5	1.7227	1.0656	1.01%	1.9636	1.4541	1.40%	1.7906	1.0530	1.00%	**1.5693**	**0.9702**	**0.93%**
+10	2.2962	1.4032	1.34%	2.4571	1.7205	1.64%	2.5636	1.5061	1.43%	**2.2672**	**1.3650**	**1.30%**
+20	2.7982	1.8130	1.73%	2.9462	1.8509	1.77%	2.7855	**1.6400**	1.63%	**2.7753**	1.6443	**1.61%**
+30	3.2849	2.0980	2.01%	3.3069	2.2141	2.11%	3.0474	1.9949	1.91%	**2.8889**	**1.7110**	**1.70%**

## Data Availability

The data that support the findings of this study are available on request from the corresponding author upon reasonable request.
